# Usability and User Experience Assessment of Health Care Conversational Agents Using Validated Subjective Instruments: Systematic Review and Comparative Analysis

**DOI:** 10.2196/102911

**Published:** 2026-07-30

**Authors:** João Pavão, Rute Bastardo, Anabela Gonçalves Silva, Nelson Pacheco Rocha

**Affiliations:** 1 Institute for Systems and Computer Engineering, Technology and Science—INESC-TEC Science and Technology School University of Trás-os-Montes and Alto Douro Vila Real Portugal; 2 UNIDCOM Science and Technology School University of Trás-os-Montes and Alto Douro Vila Real Portugal; 3 Center for Health Technology and Services Research School of Health Sciences University of Aveiro Aveiro Portugal; 4 Institute of Electronics and Informatics Engineering of Aveiro Department of Medical Sciences University of Aveiro Aveiro Portugal

**Keywords:** digital health, health care provision, usability, user experience, usability assessment, user experience assessment, conversational agents, older adults

## Abstract

**Background:**

Digital applications based on health care conversational agents (HCAs) are increasingly being developed to support health care provision. The usability and user experience of these solutions are critical determinants of their acceptability and, consequently, their impact on health-related outcomes.

**Objective:**

This systematic review aims to synthesize current evidence on the use of valid and reliable subjective instruments for assessing the usability and user experience of HCAs and to examine whether assessment outcomes vary according to their technical characteristics.

**Methods:**

A systematic search was conducted in PubMed, Web of Science, and Scopus from inception to February 2026. Studies were included if they used subjective instruments to evaluate the usability or user experience of HCAs.

**Results:**

A total of 127 studies met the inclusion criteria. The studies examined 3 categories of HCAs—text-based, voice-based, and embodied—applied to patient care, health education and prevention, health data collection, and support for daily activities among older adults. The System Usability Scale (SUS) was the most frequently used assessment instrument. Comparative analysis of SUS scores indicated higher usability ratings for text-based HCAs relative to voice-based and embodied systems. However, SUS and other subjective instruments used in the included studies may not fully capture key dimensions of usability and user experience of HCAs. Additionally, substantial heterogeneity was observed in assessment methodologies across studies.

**Conclusions:**

Comparative analysis suggested that text-based HCAs were associated with significantly higher SUS scores than voice-based and embodied HCAs. However, this finding should be interpreted with caution given the substantial heterogeneity across the included studies in health care application domains, study designs, evaluation contexts, participant populations, and HCAs’ implementation and use characteristics, as well as the limitations of the SUS in evaluating the usability of modern HCAs. The variability in assessment approaches underscores the need for standardized protocols and the development of more context-specific evaluation frameworks to enhance methodological consistency and comparability across studies.

## Introduction

Since the development of ELIZA in 1966 [1], a rule-based system developed to emulate text-based interactions with a psychotherapist, advancements in information technology have driven the widespread adoption of health care conversational agents (HCAs) [2,3], which are applications designed to support interactive, multiturn communication through written or spoken language [4]. Consumer solutions such as Siri, Google Now, Cortana, and Alexa are well-known examples of agents designed to help users find information and accomplish tasks [5].

Modern HCAs extend well beyond simple rule-based input-output interactions. Advances in large language models have facilitated their evolution from narrowly defined, task-oriented tools into open-domain systems capable of retaining conversational context, following topics across multiple turns, and sustaining coherent, continuous dialogue for more natural interactions [6,7]. In addition, AI techniques have enabled more advanced dialogue management, facilitating the exhibition of human-like behaviors, the recognition of users’ emotions and intentions, and the use of various information sources for reasoning and decision-making [8-10].

Building on these advancements, researchers have explored anthropomorphized interfaces, leading to the development of embodied agents, or virtual humans [11,12]. Unlike traditional agents, which rely solely on text or voice, embodied agents combine verbal communication with nonverbal cues (eg, posture, gestures, eye movements, or facial expressions) to foster more lifelike interactions [13-15]. This embodiment enhances users’ perception of social presence and engagement, making interactions feel more intuitive and emotionally rich [16,17]. Moreover, the introduction of virtual reality technologies has paved the way for increasingly sophisticated agents designed to promote user engagement through immersive experiences [18].

HCAs are widely used in health care [19], reflecting the widespread adoption of similar agents across other human-centered sectors, including customer service [20], entertainment [21], and education [22].

From a health care research perspective, the assessment of HCAs should consider health-related outcomes, such as symptom reduction, disease knowledge, disease self-management, and quality of life [8]. This assessment should be conducted in the context of continuous use of the applications and preferably supported by randomized controlled trials. However, before conducting randomized controlled trials, it is important to safeguard aspects that might affect health-related outcomes. This means that preliminary assessments (ie, feasibility studies) should be conducted [23] to evaluate the technical characteristics of HCAs or their quality in terms of usability and user experience and, consequently, their potential to promote user engagement among the target users [8,24].

System usability comprises several components (ie, learnability, efficiency, memorability, errors, and satisfaction) and relates to the ease of using the system interface [25]. The rise of new technologies, devices, and interaction methods has expanded the concept of usability into the broader notion of user experience, which encompasses the cognitive, affective, social, and physical aspects of interaction [26-28].

There are 3 main methods for evaluating usability and user experience, namely, inspection, testing, and inquiry [29]. The inspection method consists of a systematic analysis of interaction mechanisms carried out by experts according to specific guidelines, while the testing and inquiry methods are based on the collection of usage data [29]. The testing method involves observing users while they perform tasks with a given system to collect primarily quantitative data (eg, the number of completed tasks) that can provide empirical evidence on how to improve the interaction mechanisms [29]. By contrast, the inquiry method involves collecting qualitative data from users, namely through interviews or subjective measurement instruments such as questionnaires or scales [29]. Examples of commonly used instruments include the Post-Study System Usability Questionnaire (PSSUQ) [30] and the System Usability Scale (SUS) [31]. These instruments have had their measurement properties assessed, particularly validity (ie, how effectively usability or user experience is measured) and reliability (ie, the consistency of measurements when repeated under comparable conditions) [30-33].

In this context, this review aims to synthesize the current evidence on usability and user experience assessments of HCAs using valid and reliable subjective instruments, to identify the purposes and categories of HCAs being assessed, and to determine, through a comparative analysis, how this typification affects their usability and user experience.

Several reviews [34-41] have already focused on different aspects of HCAs (eg, the use of AI or large language models), including usability and user experience assessments (eg, [42-45]); however, these reviews did not perform comparative analyses of usability and user experience across different categories of HCAs. Such analyses are important because different characteristics of HCAs affect how users interact with them (eg, by shaping communication style, pacing, and information processing), as well as cognitive load and perceptions of trust and privacy. Therefore, this review may provide insights to inform better design decisions and the development of standardized evaluation frameworks, enabling researchers and practitioners to benchmark usability and user experience across different HCAs.

## Methods

### Study Protocol Registration and Scope

The research protocol for this systematic review was registered in the Open Science Framework and followed the PRISMA (Preferred Reporting Items for Systematic Reviews and Meta-Analyses; see Multimedia Appendix 1) guidelines [46]. The research scope (Textbox 1) was structured using the Population, Concept, and Context framework [47].

**Research scope.**

1. Population

Adults (≥18 years) managing their health conditions using digital health applications.

2. Concept

Usability and user experience assessments of health care conversational agents designed for interactive, multiturn communication through written or spoken language to support the management of users’ health conditions, supported by validated subjective instruments (ie, questionnaires or scales used to systematically collect users’ self-reported evaluations of usability or user experience).

3. Context

Community or institutional care settings.

### Literature Search

Scopus, Web of Science, and PubMed were selected as the online databases for retrieving studies included in this review. The search query included keywords representing the following 3 main components:

- HCAs: conversational agent, relational agent, virtual human, virtual agent, virtual assistant, virtual companion, virtual coach, chatbot, enhanced communication agent, embodied communication agent, ECA, and avatar.
- Health care: health care, health care, care, patient, digital health, electronic health, e-health, eHealth, older adults, and elderly.
- Usability or user experience assessment: usability, user experience, acceptance, evaluation, assessment, and test.

**Figure 1:**
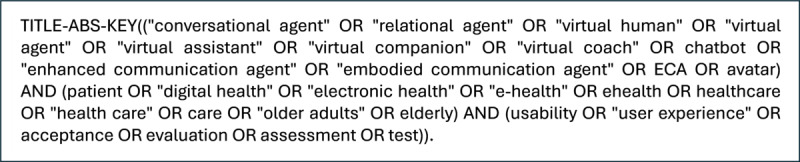
Search query for the Scopus database.

The keywords within each component were linked using the Boolean operator OR, whereas the expressions derived from different components were linked using the Boolean operator AND. As an example, Figure 1 presents the search query prepared for the Scopus database.

### Inclusion and Exclusion Criteria

To be included in the review, studies had to meet the eligibility criteria presented in Textbox 2.

**Inclusion and exclusion criteria.**

1. Inclusion criteria

- Empirical studies published in peer-reviewed scientific journals or conference proceedings.
- Adults (≥18 years).
- Studies evaluating health care conversational agents (HCAs) to support the management of users’ health conditions.
- Studies performing usability or user experience assessments of HCAs based on validated subjective instruments.

2. Exclusion criteria

- Review articles, opinion pieces, letters, and editorials.
- Children or adolescents.
- Studies evaluating the integration of HCAs into clinician-oriented digital health applications or applications focused on domains other than health care.
- Studies assessing usability or user experience without using validated subjective instruments or not focusing on this type of assessment.
- Preliminary versions of studies for which a more mature version was included in the review (eg, when a study was published as both a conference paper and later a journal article, the conference version was excluded).
- Studies published in languages other than English.
- Studies for which the full text was not available.

### Identification Process

All references retrieved from the databases were imported into a Microsoft Excel spreadsheet. The selection process then followed the general guidelines of the PRISMA flowchart [46]: (1) exclusion of duplicates, (2) title and abstract screening, and (3) full-text screening. Additionally, a backward search was performed based on the reference lists of the articles included for full-text screening.

All stages were performed manually by 2 reviewers (JP and NPR) without the use of automation tools. Disagreements between the reviewers were discussed and resolved collaboratively.

### Quality Assessment

The methodological quality assessment of the reviewed studies was conducted using a subset of the Critical Assessment of Usability Studies Scale (CAUSS) [48]. As this scale evaluates both testing and inquiry methods, the reviewers (AGS and NPR) selected 5 items related to assessments using inquiry methods: (1) representativeness of the participants, (2) proximity to the real-world context of use, (3) adequacy of the number of participants, (4) representativeness of the functionalities assessed, and (5) continuous and prolonged use of the app.

The excluded items comprised (1) items related to the validation of measurement instruments, as the eligibility criteria for this review explicitly excluded studies that did not use validated instruments; (2) items related to testing methods involving the direct observation of participants while they performed tasks to collect objective measures, such as error rates or mean task completion time; and (3) the item related to the triangulation of testing and inquiry methods, as testing methods were not considered in this review.

As only a subset of the CAUSS was applied, the resulting scores should not be interpreted as reflecting the overall quality of the usability studies, but rather the quality of the use of subjective measurement instruments.

### Data Extraction

The following data were extracted:

- Demographic characteristics, namely, the author(s), nationality of the authors’ affiliations, type of publication, and year of publication.
- Purposes of the assessed HCAs.
- Technical characteristics of the HCAs and supporting technologies (eg, AI or virtual reality).
- Types of experimental study designs (eg, cross-sectional or longitudinal studies).
- Duration of the longitudinal studies.
- Number and characteristics of the study participants.
- Recruitment and distribution of the participants.
- Validated subjective instruments assessing usability or user experience.
- Results of the use of the validated subjective assessment instruments (ie, scores and SDs).

Data were independently extracted from all included studies by 2 reviewers (JP and RB) using a customized Microsoft Excel form. Discrepancies were resolved through discussion and consensus.

### Data Synthesis

Results were synthesized into summary tables, figures, and descriptive text according to the following domains: (1) demographic characteristics (ie, publication types and publication years); (2) purposes of HCAs; (3) technical characteristics of HCAs; (4) study experimental characteristics (ie, experimental design, number and characteristics of the participants, and measurement instruments); and (5) results of usability and user experience assessments.

A quantitative analysis was performed only for studies using the SUS to assess usability. The number of studies using other instruments was insufficient for quantitative analysis. Studies using the SUS were subdivided based on the categories of HCAs being assessed. To compare SUS scores and the number of participants across the categories of HCAs, the Kruskal-Wallis test was used because the data did not follow a normal distribution (Kolmogorov-Smirnov test, *P*<.05). Medians and IQRs were provided for each group. Additionally, a subgroup analysis comparing SUS scores between studies involving older adults and those involving adults was performed for all studies and within each category of HCAs using the Mann-Whitney *U* test. The Bonferroni correction for multiple tests was applied. The significance level was set at *P*<.05. Analyses were performed using SPSS.

In addition, the effect size *r* was manually calculated as the standardized test statistic from the Mann-Whitney *U* test or the Kruskal-Wallis test (*z*) divided by the square root of the total sample size (*N*) 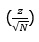 and interpreted as small (≥0.1), medium (≥0.3), or large (≥0.5) [49]. For the Kruskal-Wallis test, the ordinal eta-squared (η²*H*) was calculated as η²*H*=(*H* − *K* + 1)/(*N* − *K*), where *H* is the test statistic, *N* is the sample size, and *K* is the number of groups. It was interpreted as small (η²*H*≥0.01), medium (η²*H*≥0.06), or large (η²*H*≥0.14) [49].

Given the substantial heterogeneity among the included studies—including differences in health care application domains, study designs, evaluation contexts, participant populations, and HCAs’ implementation and use characteristics—the quantitative comparison of SUS scores was intended as an exploratory synthesis of patterns across studies rather than as evidence of causal differences in usability among HCA categories. Moreover, the SUS may not adequately capture all relevant aspects of the usability of the evaluated HCAs, such as conversational quality. Consequently, the comparative analyses should be interpreted as identifying associations at the study level rather than establishing the intrinsic usability of specific HCA categories.

## Results

### Identification of Studies

**Figure 2:**
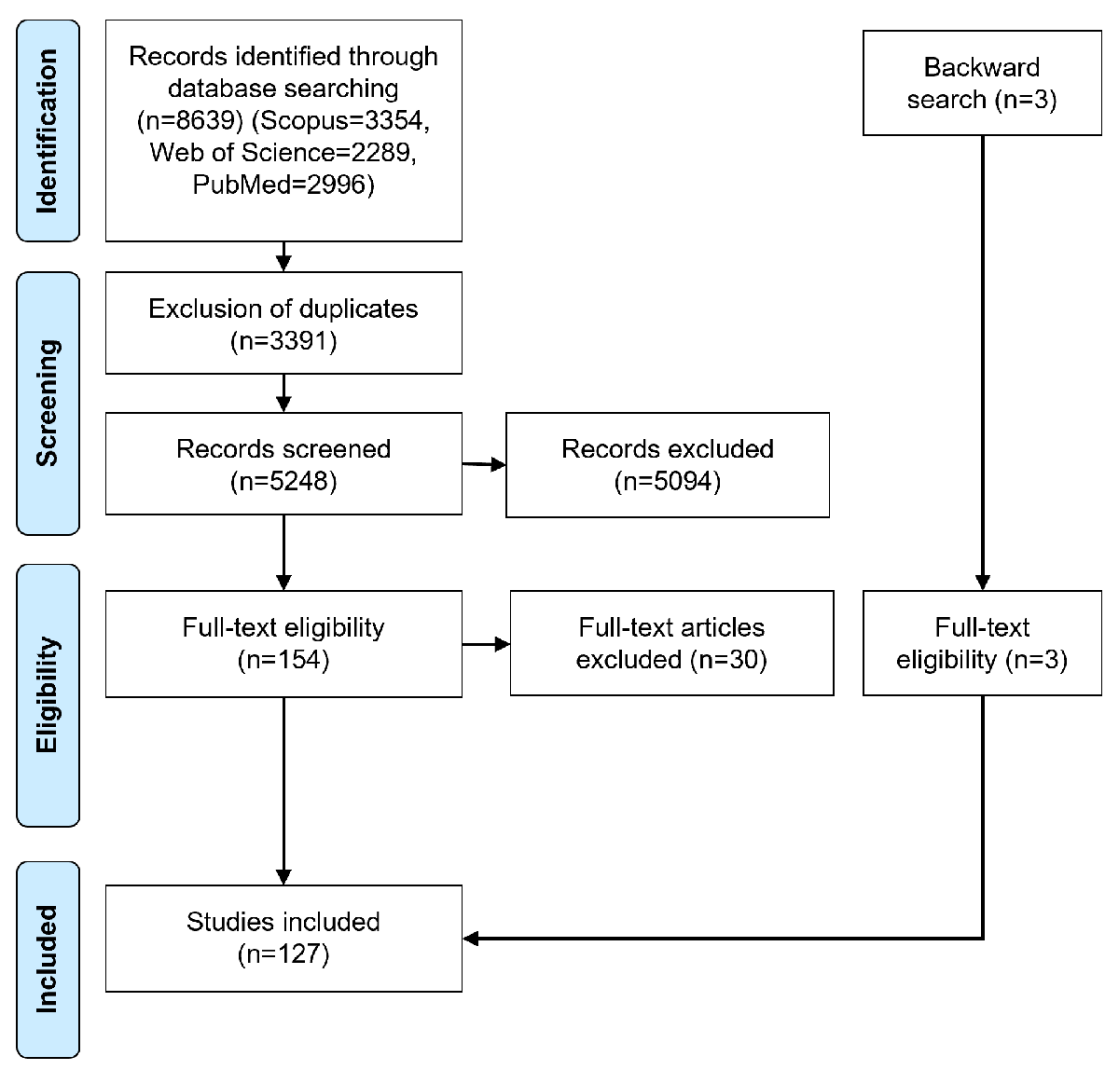
PRISMA (Preferred Reporting Items for Systematic Reviews and Meta-Analyses flowchart.

The online search was performed in February 2026, and 8639 references were retrieved. As presented in Figure 2, after the exclusion of 3391 duplicates, the titles and abstracts of 5248 references were analyzed, and 5094 were excluded according to the eligibility criteria. Therefore, 154 references were included for full-text analysis. During the full-text analysis, 30 references [50-79] were excluded because they did not meet the eligibility criteria (eg, studies that did not apply valid and reliable instruments to measure usability or user experience, or studies focused on the assessment of clinician-oriented applications). Additionally, the backward search identified 3 references, resulting in 127 references [80-206] being included in the review.

**Figure 3:**
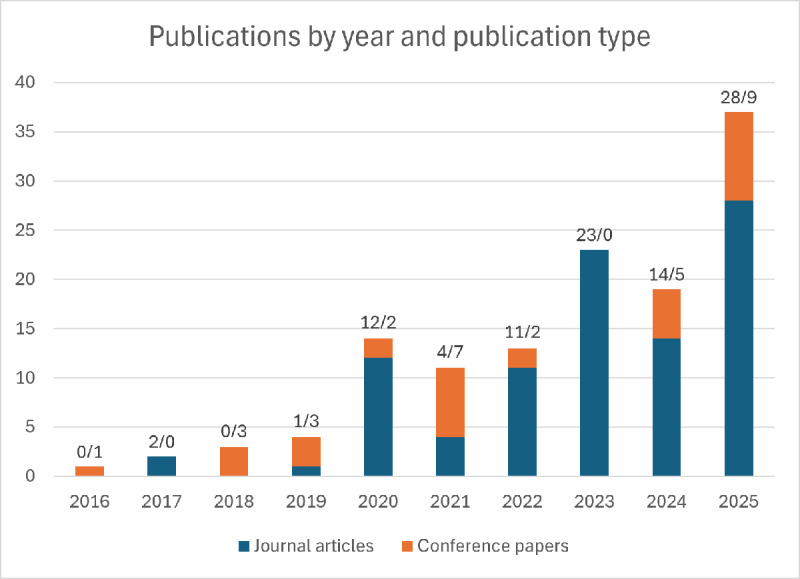
Publications by year and type.

Figure 3 presents the distribution of publication years by publication type (ie, journal articles and conference proceedings).

### Quality Assessment Results

Most CAUSS criteria considered for the methodological quality assessment were met by a majority of studies: (1) representativeness of the participants (113/127, 89%); (2) proximity to the real-world context of use (105/127, 82.7%); (3) adequacy of the number of participants (90/127, 70.9%); and (4) representativeness of the functionalities assessed (96/127, 75.6%). The exception was the criterion related to the continuous use of the application, which was met by only 44/127 (34.6%) studies. In turn, 28/127 (22%) studies met all 5 criteria.

### Purposes of the HCAs

In terms of the purposes of the HCAs described in the included articles, 4 major categories were identified:

- Patient care: 73 studies [81,82,85,87,89,92,95-97,104, 106,107,111,113-115,119,121,123,124,127,128,130, 132,133,135,136,138,140,141,144-151,153-157,159, 162,165-170,172-175,177,179-184,187-189,191,194,197,198,203-206].
- Health education and prevention: 33 studies [86,90,93,103, 109,110,112,116-118,122,125,129,131,134,137,142, 143,158,160,163,164,171,176,185,192,193,195,196,199-202].
- Health data collection: 15 studies [88,91,98,99, 101,102,105,108,120,126,139,152,161,178,186].
- Daily activities of older adults: 6 studies [80,83,84,94,100,190].

Concerning patient care, almost half of the respective articles (ie, 34 [81,82,85,92,95,97,103,104,119,121,123,124,132,147, 149,150,153,155,165-170,172,173,179,181,184,188,191,204-206]) targeted mental health disorders (eg, depression, anxiety, stress, posttraumatic stress disorder, eating disorders, and suicidal risk), while the remaining 39 [87,89,96,106,107,111,114,115,127, 128,130,133,135,136,138,140,141,144-146,148,151,154,156, 157,159,162,174,175,177,180,182,183,187,189,194,197,198,203] articles targeted a range of other conditions, including cancer [138,145,182,183,197], chronic kidney disease [144], chronic pain [115,162,198], COVID-19 [135], delirium [146], diabetes [89,96,111,115,140,194], hemophilia [136], heart-related diseases [128,130,133,154,159,174,187,203], hydrocephalus [177], intestinal ostomy [148], neurodegenerative diseases [107,141,157,175], psoriasis [127], sickle cell disease [114], traumatic injuries [106,151,180], and vestibular disorders [189].

Looking at health education and prevention, beginning with health education, some articles [117,131,137, 158,160,171,176,185,193,195,196,202] focused on general health education topics, while others focused on specific topics, such as (1) combating misinformation about COVID-19 [109,113,134], (2) endoscopy explanation [164], (3) hematologic malignancies [110], (4) hereditary breast and ovarian cancer [201], (5) perinatal information for parents [143], and (6) vaccine pharmacovigilance [142]. In the studies that focused on health prevention, the assessed HCAs were designed to promote behavior change (eg, physical activity and weight maintenance) [86,93,112,116,118,125,129,199,200], support vaccination (ie, COVID-19 [122] and human papillomavirus [192]), and provide balance training [90,163].

In terms of health data collection, 11 [88,98,101,105,108,120,126,139,152,178,186] studies assessed HCAs for collecting patient-reported outcome measures, 3 [91,99,161] focused on collecting family health history, and 1 [102] described an application designed to collect data for the evaluation of health care services and quality of care.

Finally, 6 [80,83,84,94,100,190] studies assessed HCAs designed to promote the independence and autonomy of older adults by assisting them in managing activities of daily living, including problem-solving [100] and cooking [190].

### Technical Characteristics of HCAs

Three categories of HCAs were identified: (1) text-based HCAs, (2) voice-based HCAs, and (3) embodied HCAs.

Five studies [87,98,123,153,169] assessed 2 categories of HCAs: (1) voice-based and embodied [87], (2) text- and voice-based [98,123], and (3) text-based and embodied [153,169]. The remaining studies focused on a single category, according to the following distribution:

- Text-based HCAs: 69 studies [85,86,91,99,100,102, 103,105,106,109,110,112-117,119,120,122,125,126,129-134, 137-145,147-150,154,157-159,161,164,166,168,170,177,184-187,191, 194-206].
- Voice-based HCAs: 18 studies [93,97,101,104,111,118, 124,127,135,136,146,155,165,167,172,183,190,192].
- Embodied HCAs: 35 studies [80-84,88-90,92,94-96, 107,108,121,128,151,152,156,160,162,163,171,173-176,178-182, 188,189,193].

Some studies [91,99,100,102,105,108,109,112,124,126,137,138, 147,152,155,161,163,173,178], although maintaining the HCA categories, established different comparisons, namely, (1) HCAs versus regular computer-based questionnaires [91,99,102, 105,126,161]; (2) similar applications with different functionalities [108,109,112,137,152,155,173,178] or different intervention strategies [100,124,147,163]; and (3) HCAs versus usual care [138].

**Figure 4:**
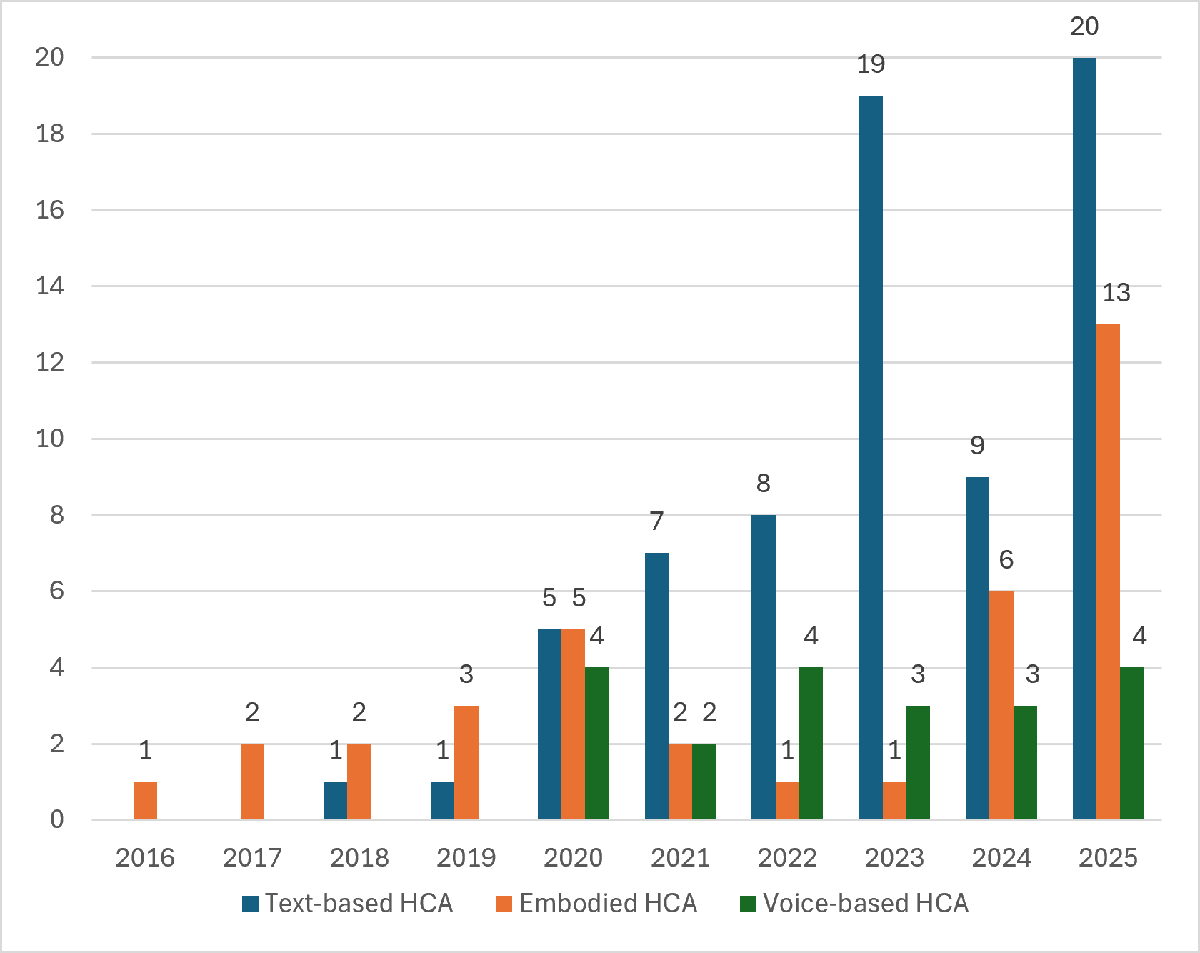
Distribution of studies across publication years, categorized by types of health care conversational agents (HCAs).

Figure 4 illustrates the distribution of studies across publication years by HCA category. According to the figure, the number of studies assessing voice-based HCAs has remained approximately constant, whereas the number of studies assessing text-based and embodied HCAs has increased over the past 3 years. Table 1 presents the relationship between HCA categories and their purposes.

**Table 1:** Relation of the categories of HCAsa and the purposes of the applications.

Purposes	Text-based HCAs	Voice-based HCAs	Embodied HCAs
Patient care—mental health	[85,103,119,123,132,147,149,150,153, 166,168-170,184,191,204-206]	[97,104,123,124,155,165,167,172]	[81,82,92,95,121,153,169,173,179, 181,188]
Patient care—other	[87,106,114,115,130,133,138,140,141,144,145, 148,154,157,159,177,187,194,197,198,203]	[111,127,135,136,146,183]	[87,89,96,107,128,151,156,162,175, 180,189]
Health care education and prevention	[86,112,113,116,122,125,129,164,185,195,196, 199-202]	[93,118,192]	[90,163,171,193]
Health data collection	[91,98,99,102,105,120,126,139,161,186]	[98,101]	[88,108,152,178]
Older adult daily activities	[100]	[190]	[80,83,84,94]

^a^HCA: health care conversational agent.

Eleven of the embodied HCAs [87,90,92,108,163,171, 174,181,182,188,189] were implemented using virtual reality technologies. Of these, 7 [92,108,163,174,181,182,189] were implemented with fully immersive virtual reality technologies, while the remaining 4 used augmented reality [90,171] and mixed reality [87,188] technologies.

Moreover, some studies [80,83,84,89,93,94,96,103,109,110, 129,134,155,156,172,179-181] implemented algorithms to recognize users’ emotional states. The main reasons for emotion recognition were to enable the HCAs to provide appropriate empathetic responses [80,83,84,89,93,94,96,109,110,129, 134,155,156,179] or to suggest specific activities for emotion regulation [155,172,180,181].

Natural language processing based on AI frameworks (eg, Google Dialogflow, Microsoft Language Understanding Intelligent Service, Rasa, or Oscova) was used in several studies to improve dialogue flow [85,86,101,110,112,117,120,124,136, 145,167,168,178,179,185,191,195-197,200,202]. In addition, large language models were used in some of the more recent studies [179,185,191,195,196,202].

AI techniques were implemented not only to improve dialogue flow, but also for other purposes, namely, (1) text-based emotion recognition [103,155]; (2) facial emotion recognition [172]; (3) definition of contextualized instructions [171]; (4) avatar generation [176]; (5) prediction of diabetes self-management [194]; and (6) improvement of the consistency and reliability of the information provided [204]. However, most studies did not report evidence of the efficacy of these techniques.

### Studies’ Experimental Characteristics

#### Experimental Design

A total of 43 studies [83,84,96,100,104,107,110,112,115, 116,118,121,122,124,127-130,132,138,140,141,144,145,149, 151,154,160,162,165,170,173,175,177,183,184,197,199-201,203-205] used a longitudinal design, among which 15 studies [100,116,121,124,138,140,141,145,162,173,175,177,184,204,205] randomized participants. The remaining 84 studies used a cross-sectional design.

Cross-sectional studies were conducted in controlled settings, including research or academic settings [81,86,87,89-91,93, 97-99,101,103,105,106,108,109,111,113,114,117,120,123,125,126, 131,133-137,139,142,143,148,152,153,155-159,161,163,166-169,171, 172,174,176,178-181,185-196,198,202], outpatient clinical settings [82,85,88,92,95,102,119,164], hospital clinical settings [146,147,150,182,206], and residential care facilities for older adults [80,94]. In the longitudinal studies, participants used the applications as part of their daily routines, although in some cases data collection was performed in controlled settings. The duration of the longitudinal studies varied from 1 week to 24 weeks: (1) 1 week, 6 [104,110,122,124,129,201] studies; (2) 2 weeks, 8 [100,121,132,151,173,183,197,204] studies; (3) 3 weeks, 1 [140] study; (4) 4 weeks, 7 [96,107,115,128,154,203,205] studies; (5) 5 weeks, 1 [199] study; (6) 6 weeks, 4 [112,160,162,165] studies; (7) 8 weeks, 2 [116,145] studies; (8) 9 weeks, 1 [170] study; (9) 12 weeks, 12 [83,84,118,127,133,138,141,144,149,175,184,200] studies; and (10) 24 weeks, 1 [177] study.

#### Number and Characteristics of the Participants

Studies included participants from specific age groups (ie, adults or older adults) and health status categories (ie, healthy participants or patients): (1) healthy adults (ie, participants between 18 and 65 years of age) [85-87,92,98,99,101,102, 104,105,108-113,117,120-122,124-126,131,132,134,137,142,143,149, 152,153,155,158,160,161,163,164,166-168,171-173,177-181,185,186,189,191, 192,195,196,199,200,202,204,205]; (2) healthy older adults (ie, participants older than 65 years) [83,84,88,90,93,94,100,115, 116,118,123,129,139,141,146,190]; (3) adult patients [81,82,89, 95,97,114,119,127,128,130,133,135,136,138,140,144,145,147,150,151,154,156, 169,170,176,183,184,187,194,197,198,201,203]; (4) older adult patients [162,165,175,182,206]; (5) healthy adults and adult patients [103,106,148,157,159,174,193]; (6) healthy adults and older adults [91,188]; and (7) healthy adults and older adult patients [80,96,107].

In terms of patients, the following conditions were identified: mental health disorders [81,82,85,92,95,97,103,104,119,121, 123,124,132,147,149,150,153,155,165-170,172,173,179,181,184,188,191,204-206], cancer [138,145,182,183,197], heart disease [154], chronic kidney disease [144], chronic pain [105,162,198], COVID-19 [95], delirium [146], dementia [107], diabetes [89,96,111,115,140,194], heart failure [130,187], hydrocephalus [177], hypertension [133,203], intestinal ostomy [148], mild cognitive impairment [141], Parkinson disease [157,175], psoriasis [127], sickle cell disease [114], stroke [128,159,174], traumatic brain injury [151], traumatic injuries [106,180], vestibular disorders [189], and other unspecified chronic diseases [87,156].

Concerning the number of participants, the studies included a total of 7040 participants, of whom 875 were older adults. However, the sample sizes varied considerably across studies. Specifically, 56 studies [82,83,87,91,92,94,95,98-102,107,109, 112-116,121,122,124,126,130,134-142,144,145,149,152,153, 158,160,161,167,169,170,173,177,180,181,191,194,195,197,200,203,204,206] included more than 30 participants, and 18 of these studies [82,95,100,112,126,139,142,149,158,160,161,170,173,180,181,194,200,204] enrolled more than 100 participants. The remaining 71 studies included 30 or fewer participants.

#### Validated Subjective Assessment Instruments

Three categories of validated subjective instruments were identified:

- Usability assessment instruments: (1) PSSUQ [30], (2) SUS [31], (3) Chatbot Usability Questionnaire (CUQ) [207], (4) Computer System Usability Questionnaire (CSUQ) [208], (5) mHealth App Usability Questionnaire (MAUQ) [209], (6) Usability Metric for User Experience—Lite Version (UMUX-Lite) [210], and (7) Usefulness, Satisfaction, and Ease of Use Questionnaire (USE) [211].
- User experience assessment instruments: (1) User Experience Questionnaire (UEQ) [212] and (2) User Experience Questionnaire—Short Version (UEQ-S) [213].
- Acceptability assessment instrument: Acceptability E-Scale (AES) [214], which includes a subscale for assessing usability.

Nine studies [86,105,117,119,145,146,174,175,191] applied more than 1 assessment instrument: (1) UEQ and SUS [86,105,117,145,174,175,191]; (2) SUS and USE [119]; and (3) CUQ and SUS [146]. See Table 2 for the validated subjective assessment instruments.

**Table 2:** Validated subjective assessment instruments.

Instruments	Studies	Total, n
System Usability Scale	[80,81,83-90,92,96-98,100,105-109,111-123,126-130,132,133,135,136, 140-142,144-148,150,151,153-157,160-168,170-172,174-177, 179,180,182,184,186,190-202]	91
User Experience Questionnaire	[86,101-103,105,110,117,143,145,174,175,178,181,189,191]	15
Chatbot Usability Questionnaire	[125,134,138,146,203,204]	6
Computer System Usability Questionnaire	[91,99,131,139,205]	5
Acceptability E-Scale	[82,95,149,169,206]	5
mHealth App Usability Questionnaire	[159,173,185,187]	4
Usefulness, Satisfaction, and Ease of Use Questionnaire	[94,104,119,183]	4
User Experience Questionnaire—Short Version	[93,152,158]	3
Usability Metric for User Experience—Lite Version	[124,188]	2
Post-Study System Usability Questionnaire	[137]	1

### Results of Usability and User Experience Assessments

The usability and user experience scores were generally high. The PSSUQ was applied in only 1 [137] study, which reported a total score of 6.0 (above average, ≥6). Additionally, Table 3 summarizes the ranges of usability and user experience scores for the remaining instruments, based on the studies that reported these scores.

**Table 3:** Range of the reported usability and user experience scores according to subjective assessment instruments and the standardized scores for each instrument indicating good usability.

Instruments	Score range	Standardized scores indicating good usability or user experience
System Usability Scale	51.4-93.8	≥70.0
Chatbot Usability Questionnaire	40.6-85.9	≥70.0
Computer System Usability Questionnaire	4.6-5.8	≥4.5
Acceptability E-Scale	22.0-25.4	≥24.0
mHealth App Usability Questionnaire	4.8-6.7	≥5.0
Usefulness, Satisfaction, and Ease of Use Questionnaire	4.5-5.7	≥5.0
Usability Metric for User Experience—Lite Version	82.4-86.1	≥70.0
User Experience Questionnaire and User Experience Questionnaire—Short Version	0.4-1.7	≥1.0

### Comparative Analysis of the SUS Results

The following comparative analysis should be interpreted as exploratory because it synthesizes SUS scores reported by independent studies rather than direct comparisons conducted under common experimental conditions. Consequently, the observed differences among HCA categories may be influenced not only by the interaction modality itself but also by variations in health care application domains, study designs, evaluation contexts, participant populations, and HCA implementation and use characteristics. In addition, although the SUS is a well-established measure of perceived usability, it may not adequately capture important aspects of HCAs, such as conversational quality, which is influenced by factors including turn-taking, the ability to sustain coherent and continuous dialogue, and the naturalness of interactions.

**Figure 5:**
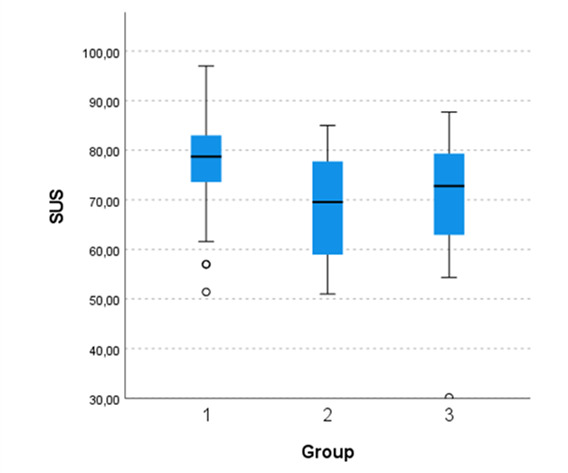
Median and IQR for System Usability Scale (SUS) scores (group 1, text-based HCAs; group 2, voice-based HCAs; and group 3, embodied HCAs). HCA: health care conversational agent.

Considering the studies that applied the SUS and reported usability scores, a significant difference among groups was found when comparing usability scores across the 3 HCA categories (*P*=.001; η²*H*=0.11; Figure 5, Table 4). Pairwise comparisons showed significant differences between text-based and embodied HCAs (*P*=.02) and between text- and voice-based HCAs (*P*=.006). No significant difference was found between voice-based and embodied HCAs (*P*>.99). When comparing the number of participants across the 3 HCA categories, no significant difference was found (*P*=.18; η²*H*=0.016).

**Table 4:** Comparative analysis of SUS^a^ scores across the 3 categories of HCAs^b^.

Category	Number of studies, n	Number of participants, median (IQR^c^)	SUS scores, median (IQR^c^)	SUS range
Text-based HCAs	58	30.00 (11.50-52.75)	78.70 (73.43-83.06)	51.40-97.00
Voice-based HCAs	18	22.00 (13.0-34.25)	69.55 (58.90-78.47)	51.00-85.00
Embodied HCAs	26	18.00 (11.00-26.25)	72.79 (62.29-79.38)	30.13-87.70

^a^SUS: System Usability Scale.

^b^HCA: health care conversational agent.

^c^25th-75th percentiles.

**Figure 6:**
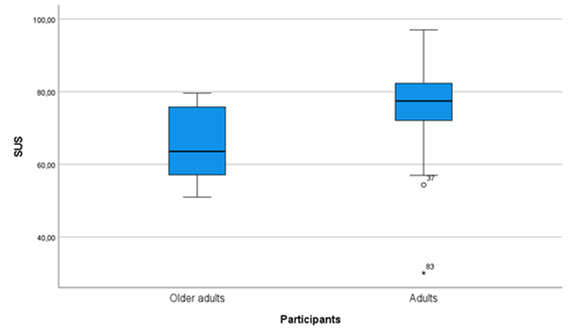
Median and IQR for System Usability Scale (SUS) scores in studies using adults and older adults.

A significant difference in SUS scores was found when comparing studies involving adults with those involving older adults in the overall sample of studies (*P*<.001; *r*=0.39; Figure 6, Table 5). Similarly, a significant difference in SUS scores between studies involving older adults and those involving adults was found for text-based HCAs (*P*=.009; *r*=0.33), indicating lower SUS scores in studies involving older adults. No significant between-group difference was found for voice-based HCAs (*P*=.10; *r*=0.38) or embodied HCAs (*P*=.20; *r*=0.25).

**Table 5:** Comparative analysis of SUS^a^ scores across age range (adults vs older adults).

Category and age group			Number of studies, n		SUS, median (IQR^b^)
**All HCAs^c^**					
	Adults	82		77.45 (72.06-82.43)	
	Older adults	19		63.56 (57.07-76.19)	
**Text-based HCAs**					
	Adults	51		79.50 (75.00-83.80)	
	Older adults	6		64.05 (55.58-78.28)	
**Voice-based HCAs**					
	Adults	13		72.10 (59.97-81.17)	
	Older adults	5		59.69 (52.17-72.00)	
**Embodied HCAs**					
	Adults	18		74.41 (67.36-81.08)	
	Older adults	8		63.75 (57.65-77.78)	

^a^SUS: System Usability Scale.

^b^25th-75th percentiles.

^c^HCA: health care conversational agent.

## Discussion

This review provides an overview of the current evidence on the usability and user experience of HCAs assessed using validated subjective instruments. As HCAs become increasingly widespread, a growing number of individuals interact with them daily, and it is anticipated that these agents will play an important role in future interactive systems. Therefore, it is crucial to investigate the assessment of their usability and user experience, particularly in the context of emerging human-machine interaction, because well-designed assessments contribute to the overall improvement of interactions. Although well-designed interactions do not necessarily mean that digital health applications are effective, their efficacy and effectiveness may be limited by usability and user experience constraints [215].

Patient care emerged as the predominant application domain for HCAs, particularly in mental health. Other reviews [8,216] have already identified the importance of patient care, while the efficacy of HCAs in supporting mental health disorders has been the subject of several systematic reviews (eg, [217,218]) and a meta-analysis (eg, [219]). Among the other application domains addressed, HCAs may support health education and prevention by optimizing the delivery of understandable, personalized health education at scale and by providing interactive and engaging information across platforms (eg, mobile, web, or smart devices) [220,221]. Similarly, health data collection has already been identified as a core use case of HCAs [216,222], namely, in terms of automating diagnostic interviews and longitudinal monitoring of patient-reported outcomes (eg, anxiety or depression scales) to track symptoms and adherence to treatments over time. Furthermore, the use of HCAs designed to promote the independence and autonomy of older adults by assisting them in managing activities of daily living and self-management of health conditions is in line with current interest in developing digital applications to support the care and well-being of older adults [223,224]. Another relevant point is that a high proportion of the studies included older adults, who accounted for 875 of the 7040 (12.43%) participants.

Although text-based HCAs were the most frequently evaluated, all 3 identified categories were applied across different health care domains. This suggests that developers select interaction modalities according to technological feasibility and user requirements rather than the clinical purpose itself.

The increasing integration of virtual reality with embodied HCAs reflects growing interest in immersive human-computer interaction. However, the relatively limited number of studies indicates that these technologies remain at an early stage of adoption in health care, likely due to higher development costs, hardware requirements, and implementation complexity.

Although AI was increasingly incorporated into HCAs, most of these implementations appear to remain at a relatively immature stage, which is consistent with other studies demonstrating the difficulties of translating AI implementations into clinical workflows [225-227].

SUS clearly dominated the assessment of HCAs, despite having been developed for generic interactive systems. SUS is widely used for usability evaluation of different types of digital health applications [215,228-230] because it is an easy and quick tool with a scoring system that has been translated into multiple languages [228]. However, it was not originally developed to assess complex multimodal interaction mechanisms and does not capture the holistic user experience because it focuses only on usability aspects such as ease of use, satisfaction, and learnability.

Although several alternative instruments were identified, CUQ was the only instrument specifically appropriate for assessing HCAs, even though it does not fully capture the multimodal and AI-driven characteristics of modern HCAs. The absence of recently developed instruments [46], such as the Conversational Agents Scale [231], the Conversational Agent’s Usage Scale [232], the User Experience Evaluation of Conversational Agents [233], or the Conversational Agent Scale for User Experience [234], further highlights the need for greater methodological standardization in future evaluations. Moreover, a relevant number of studies were excluded from this review because usability and user experience assessments were based on questionnaires consisting of Likert scale questions developed by the researchers. However, these questionnaires were not validated instruments because they had not undergone psychometric analysis, which is frequently a lengthy process that can be particularly challenging for researchers who lack experience in developing and validating subjective instruments.

Usability and user experience assessments were conducted in both controlled (ie, research or academic settings, outpatient clinical settings, hospital clinical settings, and institutional residences for older adults) and uncontrolled (eg, participants’ homes) environments. Controlled experiments generally focus on intensive sessions to capture immediate usability or user experience feedback. These experiments enable consistent data collection, but they may constrain the observation of more natural interaction patterns. By contrast, longitudinal studies conducted in uncontrolled settings allow participants to engage with HCAs more organically, integrating their use into daily routines. Therefore, uncontrolled environments provide authentic user experiences and valuable insights, as well as functional challenges, including connectivity issues, reflecting authentic usage scenarios. Moreover, many longitudinal studies strategically combined uncontrolled environments with data collection in controlled environments.

The participants comprised both patients and healthy adults or older adults. However, the reviewed studies often did not adequately justify their choice of sample sizes. Small samples can undermine the reliability of results by increasing the risk of sampling bias and reducing statistical power. Consequently, the findings may not capture the full variability of the population, thereby restricting their generalizability.

The quality assessment of the components related to the application of subjective measurement instruments highlighted methodological limitations, including insufficient evaluation of prolonged real-world use and inadequate justification of sample sizes, the 2 CAUSS items with lower percentages. These shortcomings reduce confidence in the ecological validity and generalizability of the reported usability findings.

Looking at the assessment results, the usability and user experience of HCAs tend to be above average; however, the results indicate differences among the various HCA categories. The comparison of usability scores across the 3 HCA modalities revealed statistically significant differences, with pairwise comparisons indicating that text-based HCAs achieved significantly higher usability scores than both voice-based and embodied HCAs, whereas no significant differences were observed between voice-based and embodied HCAs. The estimated eta-squared (η²*H*=0.11) indicates a moderate effect size, suggesting a moderate association between interaction modality and users’ perceptions of usability. No significant differences were found in the number of participants across the 3 HCA categories, indicating that the studies included broadly comparable sample sizes. Consequently, systematic differences in study size are unlikely to fully explain the observed differences in usability.

Several factors may contribute to the higher usability observed for text-based HCAs. Although the reviewed studies did not directly investigate the underlying mechanisms, one possible explanation is users’ greater familiarity with text-based communication, particularly through messaging applications. Moreover, text interfaces provide a persistent conversation history that enables users to review previous exchanges, potentially increasing their sense of control and reducing memory demands during interaction. In addition, text-based interactions allow users to engage with HCAs at their own pace, giving them more time to read, reflect on, and formulate responses. By contrast, voice-based HCAs may be affected by speech recognition inaccuracies, environmental noise, and privacy concerns, whereas embodied HCAs introduce additional visual and social interaction elements that may increase interaction complexity without necessarily improving perceived usability.

The results also suggest that older adults tend to perceive HCAs as less usable than adults. This may be associated with older adults generally having lower digital literacy, as well as age-related accessibility needs. For example, age-related sensory, cognitive, and motor changes, together with the higher prevalence of multimorbidity among older adults, may affect their ability to interact effectively with HCAs. Nevertheless, the available evidence from the included studies does not allow this explanation to be confirmed.

Interestingly, the difference between adults and older adults was statistically significant only for text-based HCAs, despite this modality achieving the highest usability scores overall. A potential explanation is that text-based HCAs are currently more widespread than voice-based or embodied HCAs, which may lead to greater differences in familiarity and prior experience between age groups.

Although no statistically significant age-related differences were observed for voice-based or embodied HCAs, the estimated effect sizes were of comparable magnitude across modalities. Therefore, the absence of statistical significance may reflect the smaller number of available studies and limited statistical power rather than provide evidence that age has no influence on perceived usability in these interaction modalities.

These results should be interpreted with caution. The included studies exhibited substantial heterogeneity in terms of application domains (patient care, health education and prevention, health data collection, and support for daily activities), study designs (eg, cross-sectional or longitudinal), evaluation contexts (controlled versus real-world settings), target populations, underlying technologies (eg, AI, large language models, or virtual reality), task complexity, and duration of HCA use (single-session vs prolonged use). Consequently, the observed differences in SUS scores may have been influenced by factors beyond the interaction modality itself. Furthermore, the SUS assesses perceived usability rather than objective performance or long-term acceptance, and therefore high SUS scores do not necessarily translate into sustained engagement or continued use. Future studies should investigate which specific design characteristics contribute most to usability across different HCA modalities while accounting for user characteristics and contextual factors.

Despite the use of extensive search strategies to locate relevant studies, some articles may have been overlooked due to variations in terminology. Furthermore, the exclusion of non-English publications and gray literature may have limited the overall representativeness of the findings and introduced language and publication bias, as studies published in other languages or unpublished studies with null or negative findings may have been underrepresented.

The wide variety of assessment instruments used meant that it was generally not possible to identify enough studies to enable a comparative analysis of usability or user experience outcomes by instrument across different HCA categories. The only exception was SUS; however, as it was developed in the 1990s, it may not adequately capture relevant aspects of the usability of modern HCAs.

Finally, this review focused solely on the usability and user experience of HCAs and did not address their efficacy or effectiveness.

In conclusion, this review included 127 studies applying validated subjective instruments to assess the usability and user experience of 3 HCA categories: text-based, voice-based, and embodied HCAs. In terms of health care applications, HCAs aimed to support patient care, health education and prevention, health data collection, and daily activities of older adults.

The usability and user experience of HCAs were assessed in cross-sectional and longitudinal studies using the following instruments: SUS, UEQ, UEQ-S, CUQ, AES, MAUQ, UMUX-Lite, USE, CSUQ, and PSSUQ.

This review highlights that, except for CUQ, the identified subjective assessment instruments were not specifically developed to evaluate the usability and user experience of HCAs. Therefore, the findings suggest that such evaluations may be limited by the inadequacy of these instruments.

Considering the comparative analysis of SUS scores, text-based HCAs tend to show higher usability scores than voice-based and embodied HCAs, and older adults tend to perceive HCAs as less usable than adults. However, these findings represent associations observed across the included studies rather than direct evidence that one HCA category is inherently more usable than another, because the studies differ substantially in their health care applications, design, evaluation contexts, participant populations, and implementation and use characteristics. Moreover, a relevant number of studies used small sample sizes, which reduce statistical power, may fail to capture population variability, limit the generalizability of the results, and increase the risk of misleading conclusions. Most HCAs were also assessed in controlled environments at a single point in time, without considering the impact of continuous and long-term use in real-world settings.

Considering the substantial differences in how evaluations were designed and conducted across studies, especially regarding data collection methods, participant selection, testing conditions, and reporting of results, there is a need for standardized protocols and established guidelines to measure the usability and user experience of HCAs. Such standardization would improve methodological consistency and enhance the comparability of findings across studies, enabling researchers and practitioners to benchmark usability and user experience.

## Appendix

Multimedia Appendix 1 PRISMA (Preferred Reporting Items for Systematic Reviews and Meta-Analyses) 2020 checklist.

## Abbreviations

AES: Acceptability E-Scale
CAUSS: Critical Assessment of Usability Studies Scale
CSUQ: Computer System Usability Questionnaire
CUQ: Chatbot Usability Questionnaire
HCA: health care conversational agent
MAUQ: mHealth App Usability Questionnaire
PRISMA: Preferred Reporting Items for Systematic Reviews and Meta-Analyses
PSSUQ: Post-Study System Usability Questionnaire
SUS: System Usability Scale
UEQ: User Experience Questionnaire
UEQ-S: User Experience Questionnaire—Short Version
UMUX-Lite: Usability Metric for User Experience—Lite Version
USE: Usefulness, Satisfaction, and Ease of Use Questionnaire
